# Enteropathogenic and enterohemorrhagic *E. coli*: ecology, pathogenesis, and evolution

**DOI:** 10.3389/fcimb.2013.00015

**Published:** 2013-04-30

**Authors:** Elizabeth L. Hartland, John M. Leong

**Affiliations:** ^1^Department of Microbiology and Immunology, University of MelbourneParkville, VIC, Australia; ^2^Murdoch Children's Research Institute, Royal Children's HospitalParkville, VIC, Australia; ^3^Department of Molecular Biology and Microbiology, Tufts University School of MedicineBoston, MA, USA

The gastrointestinal pathogens enteropathogenic and enterohemorrhagic *E. coli* (EPEC and EHEC) continue to pose a threat to human health worldwide. While EPEC remains a significant cause of diarrhea in low-income countries, EHEC is more common as a food or water-borne pathogen in industrialized countries. A major difference between EPEC and EHEC is that EPEC has only a human reservoir of infection while EHEC is a zoonotic disease. Strains of EHEC are commensal in many ruminants, particularly cattle, and hence entry into the food chain through fecal contamination of food or water is a risk factor for infection. Another characteristic of EHEC but not EPEC is the production of Shiga toxins, which are associated with the development of severe complications of infection, namely hemorrhagic colitis (HC) and the hemolytic uremic syndrome (HUS). Whereas, HUS can affect patients of any age, EPEC remains a pathogen of infants less than 2 years of age (Robins-Browne and Hartland, 2002).

Despite their differing epidemiology and clinical manifestations, EPEC and EHEC are highly related and share many virulence determinants and features. Both pathogens share a distinctive mechanism of intestinal colonization known as attaching and effacing A/E lesion formation. A/E lesions are characterized by tight attachment of the bacteria to the enterocyte surface, the localized destruction of microvilli and massive ultrastructural changes underneath the adherent bacteria resulting from the accumulation of filamentous actin (Wong et al., 2011). The locus of enterocyte effacement (LEE) is essential for A/E lesion formation, and encodes a type III section system (T3SS) that translocates multiple effector proteins into the infected enterocyte. The LEE is quintessential for the definition of EPEC, as all strains of EPEC carry LEE and induce A/E lesions. The LEE is also used to define some Shiga-toxin producing *E. coli* (STEC) as EHEC such as O157:H7, although it role in defining EHEC is blurred somewhat by the existence of STEC that lack LEE yet cause HC and HUS and, as in the case of the recent EHEC O104:H4 outbreak, may be highly virulent (Frank et al., 2011). For true A/E pathogens, the LEE is absolutely required for infection and in EHEC O157:H7 its expression appears to be enhanced by passage in the mammalian gut (Brady et al., 2011), suggesting that animal-to-person or person-to-person transmission may be under positive selection during acute infection.

The LEE T3SS system is similar to those found in other pathogens but has an additional filament to the T3SS needle made of polymerized EspA subunits. EspA is essential for translocation to occur and may also provide some adhesive function during bacterial colonization. The most widely accepted model is that the hydrophobic LEE-encoded proteins, EspB and EspD, form a pore in the host cell membrane that provide a conduit for effector translocation through the T3SS. Together EspA, EspB, and EspD constitute the LEE translocon. A recent proposal is that type III secretion is a two-step process whereby surface-localized effector proteins are part of an intermediate effector-translocator complex that precedes effector translocation (Pilar and Coombes, 2011). Future work will be required to reconcile this model with the presence of the EspA filament that structural evidence suggests directly attaches to the T3SS needle (Sekiya et al., 2001).

Among the many translocated effector proteins, Tir plays a critical role in intimate attachment through binding the outer membrane protein intimin. Although intimin and Tir alleles are largely interchangeable despite sequence variation among different A/E pathogens, intimin has been associated with additional Tir-independent adhesive functions. This has also been explored as a source of EPEC/EHEC tissue tropism in the past (Fitzhenry et al., 2002). Here a Tir-independent role for intimin in the colonization of streptomycin-treated mice provides more evidence that in some experimental systems, intimin promotes intestinal colonization independently of its role in Tir-binding (Mallick et al., 2012).

While the intimin-Tir interaction has been intensely studied, not all effector proteins contribute to A/E lesion formation and cytoskeletal changes. It has recently emerged that several effector proteins play a role in dampening the inflammatory response. The effector proteins involved are all encoded outside the LEE and have novel enzymatic functions that target NF-κB and MAPK signaling. For example, NleE is a cysteine methyl transferase that methylates the zinc finger domain of TAB2/3, thereby inhibiting TAB2/3 interaction with ubiquitinated TRAF and blocking the phosphorylation of IκB by the IKK complex and hence the degradation of IκB (Newton et al., 2010; Zhang et al., 2011). NleC and NleD are zinc metalloproteases that cleave the p65 subunit of NF-κB and the MAPKs p38 and JNK, respectively (Baruch et al., 2011; Pearson et al., 2011). NleH has both anti-apoptotic and anti-inflammatory effects acting in part by binding ribosomal protein S3 (Hemrajani et al., 2010; Gao and Hardwidge, 2011).

Although much research is aimed at understanding the function of the LEE and the translocated effector proteins [recently reviewed in Wong et al. (2011)], the carriage and transfer of virulence genes among different *E. coli* pathogens makes the study of other potential virulence factors critically important. For example, EhaJ is an autotransporter protein that is shared by strains of belonging to both EPEC and EHEC lineages. Autotransporters are highly prevalent in EPEC/EHEC genomes, and many contribute to inter-bacterial interactions and biofilm formation (Wells et al., 2010). EhaJ also has biofilm-producing properties when expressed in laboratory strains of *E. coli*, and this requires the function of a putative glycosyltransferase encoded by the adjacent gene, *egtA*. In addition, EhaJ has extracellular matrix binding properties, suggesting that the autotransporter may contribute to host infection. Other factors apart from the T3SS may also contribute to the ability of EHEC O157:H7 to persist and colonize the surface of plants, including pili and flagella (Saldana et al., 2011), as occurred in a large outbreak of EHEC O157:H7 that was spread by contaminated spinach (Wendel et al., 2009).

The reservoir of EHEC in cattle deserves particular attention as many LEE-independent factors, such as autotransporters, may contribute to persistence in this natural host. Indeed recent work reported here on the interactions of EHEC O157:H7 with bovine rectal epithelial cells suggests that the bacteria are internalized though an intimin- and Tir-independent mechanism. The rectum is the main site of EHEC O157:H7 colonization in cattle and acts as an ongoing source of contamination through fecal shedding (Low et al., 2005). To identify factors that contribute to the ability of EHEC to attach to rectal epithelial cells, here Bai et al. screened BAC clones derived from EHEC O157:H7 in a competition-based assay. They identified fimbriae and the autotransporter EhaA as candidates for EHEC-bovine epithelium interactions (Bai et al., 2011).

Because of its prominence in large outbreaks of disease, much research has focused on EHEC O157:H7 virulence. Nevertheless non-O157 EHEC, of which there are more than 100 serotypes, also have the capacity to cause HUS and we know far less about their genome structure and evolution (Coombes et al., 2011). As this information accumulates, we will have a much greater understanding of the relationship between EHEC/EPEC and other *E. coli* pathogens that all contribute to a common gene pool from which new pathogens, such as EHEC O104:H4, may emerge. Ongoing surveillance with high throughput genomics and molecular and infection studies are the keys to understanding the ecology and pathogenesis of EPEC and EHEC and are our only weapons against a fast evolving and dynamic group of pathogens.
